# The complete plastome of *Andreaea rupestris* Hedw. (Andreaeaceae, Bryophyta)

**DOI:** 10.1080/23802359.2021.1920507

**Published:** 2021-05-17

**Authors:** Xin-Jie Jin, Ling-Juan Liu, Sheng-Long Liu, Zhi-Xin Zhang, Rui-Liang Zhu

**Affiliations:** a Bryology Laboratory, School of Life Sciences, East China Normal University, Shanghai, China; b Fengyangshan Administrative Office, Fengyangshan-Baishanzu National Nature Reserve, Zhejiang, China

**Keywords:** Granite moss, lantern moss, plastome, phylogenomics

## Abstract

*Andreaea rupestris* Hedw., one of the lantern mosses, is the lectotype of the genus *Andreaea* Hedw. (Andreaeaceae). Here we present its complete plastome. The plastome of *A. rupestris* is successfully assembled from raw reads sequenced by HiSeq X ten system. Its total length is 135,214 bp consisting of four regions: large single copy (LSC) region (92,780 bp), small single copy (SSC) region (21,102 bp), and two inverted repeat regions (IRs; 10,666 bp per each). It contains 134 genes (88 coding genes, 8 rRNAs, and 38 tRNAs). The overall GC content is 30.3% and in the LSC, SSC, and IR regions are 27.5%, 26.5%, and 46.2%, respectively. The present data will be an important sequence resource for further studies on the important early diverging lineage of mosses.

*Andreaea* Hedw., the type genus of the granite moss family Andreaeaceae, is commonly known as lantern moss due to the appearance of dehisced sporangia (Hedwig 1801; Schofield 1985). The plants of *Andreaea* are usually dark in color, varying from dark red/brown/green to black depending on its life stage. *Andreaea rupestris* is the type species of the genus *Andreaea* Hedw. (Andreaeaceae). The first mitochondrial genome sequence of Andreaeaceae was presented by Huang et al. (2019) based on *Andreaea wangiana* P.C.Chen known only from China. Recent molecular phylogenetic studies presented that Andreaeaceae is an early divergence lineage in the evolution of mosses (Liu et al. 2019). Till now, there is no complete plastome of Andreaeopsida. Here, we present the plastome of *A. rupestris* as the first plastome of *Andreaea* as well as class Andreaeopsida.

*Andreaea rupestris* was collected in the Huangmaojian peak of Fengyangshan Nature Reserve, Zhejiang, China (27°53′21″N, 119°11′14″E). The specimen was deposited at the herbarium of East China Normal University (HSNU, http://museum.ecnu.edu.cn/; Rui-Liang Zhu, rlzhu@bio.ecnu.edu.cn) under the voucher number Zhu & Zhang 20200723-14. DNA was extracted using DNA Plantzol Reagent (Hangzhou LifeReal Biotechnology Co., Ltd, Hangzhou, China). Genome sequencing was performed using HiSeq X ten system at BGI (Shenzhen), China, and *de novo* assembly was done by GetOrganelle pipeline (Jin et al. 2020). Geneious version 11.0.3 (Kearse et al. 2012) was used for plastome annotation, with *Takakia lepidozioides* S.Hatt. & Inoue plastome (AP014702) as a reference. CPGAVAS2 was used to further verify the tRNA genes (Shi et al. 2019).

The plastome of *A. rupestris* (GenBank accession no. MW561627) is 135,214 bp long (GC ratio is 30.3%) and has four subregions: 92,780 bp of large single copy (LSC; 27.5%) and 21,102 bp of small single copy (SSC; 26.5%) regions separated by 10,666 bp of inverted repeat (IR; 46.2%). It contains 134 genes (88 protein-coding genes, 8 rRNAs, and 38 tRNAs); 9 genes (4 rRNAs and 5 tRNAs) duplicated in IR regions.

Fifteen complete plastomes including *A. rupestris* were used for Bayesian Inference (BI, number of generations is 2,000,000) and maximum-likelihood (ML, bootstrap repeat is 1000) phylogenic trees using MRBAYES v3.2.7 (Ronquist and Huelsenbeck 2003) and IQ-TREE v2.0.6 (Nguyen et al. 2015), respectively, after aligning whole plastome using MAFFT v7.149b (Katoh and Standley 2013).

Our analyses show the basal groups of mosses as a paraphyletic assemblage, with the *Sphagnum palustre* L. and *Flatbergium sericeum* (Müll. Hal.) A.J.Shaw (Shaw et al. 2016) splitting off first, followed by a well-supported *A. rupestris*. Our trees also support a sister-group relationship between class Andreaeopsida (*A. rupestris*) and the rest of crown mosses as shown by Liu et al. (2019) (Figure 1). With the help of next-generation sequencing technology, more and more complete plastome of mosses will be published in the near future, which will allow us to have a better understanding of their phylogenetic relationships.

**Figure 1.**
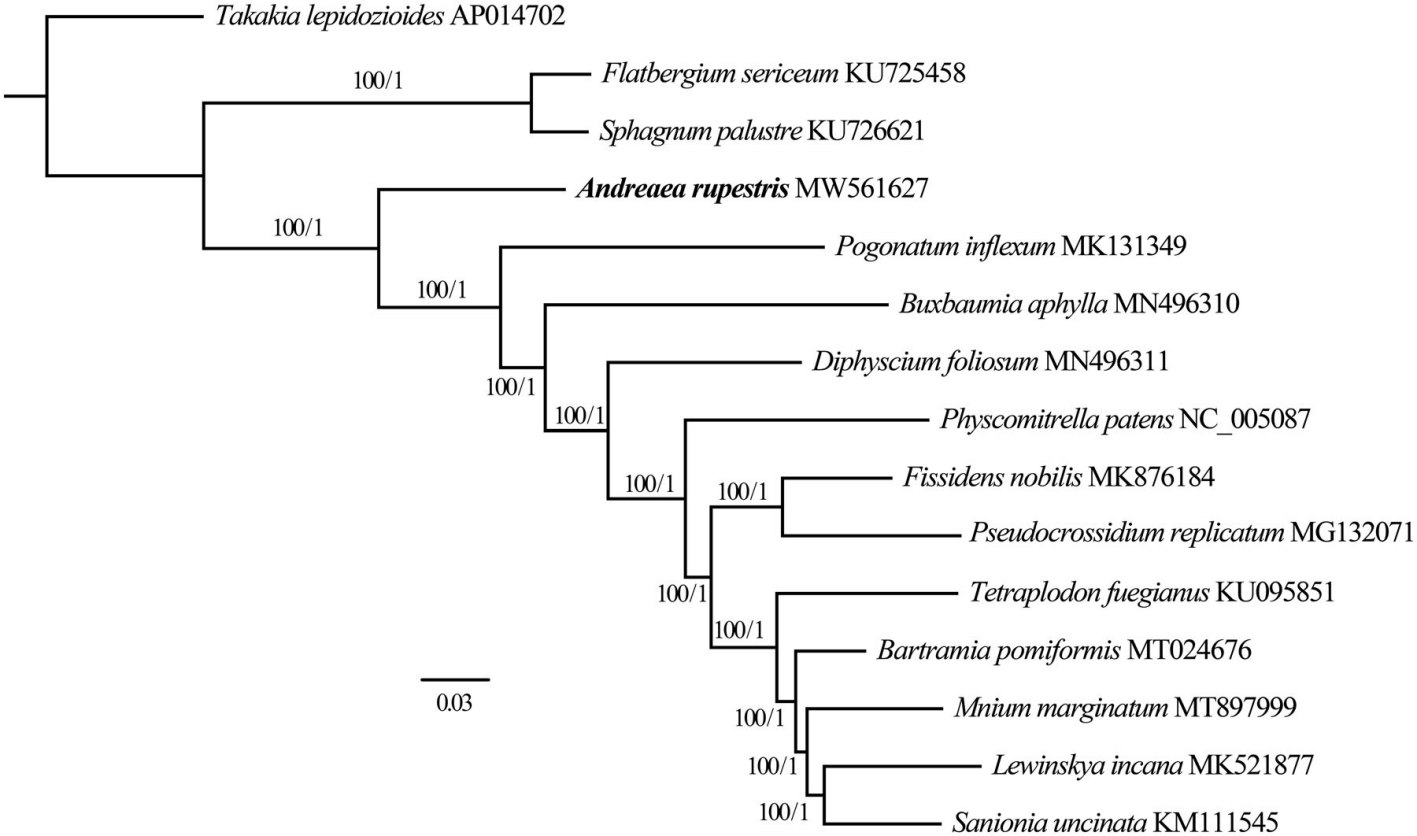
Maximum-likelihood (ML) and Bayesian inference (BI) phylogenetic tree of 15 complete chloroplast genomes: *Andreaea rupestris* (MW561627, in this study), *Bartramia pomiformis* (MT024676), *Buxbaumia aphylla* (MN496310), *Diphyscium foliosum* (MN496311), *Fissidens nobilis* (MK876184), *Flatbergium sericeum* (KU725458), *Lewinskya incana* (MK521877), *Mnium marginatum* (MT897999), *Physcomitrella patens* (NC_005087), *Pogonatum inflexum* (MK131349), *Pseudocrossidium replicatum* (MG132071), *Sanionia uncinata* (KM111545), *Sphagnum palustre* (KU726621), *Takakia lepidozioides* (AP014702), *Tetraplodon fuegiuanus* (KU095851). The ingroup consisted of 14 moss species representing 13 orders and 5 classes and Takakia lepidoziodes (AP014702) as an outgroup. Phylogenetic tree was drawn based on the ML tree. The numbers above branches indicate bootstrap values (BS) and Bayesian posterior probabilities (PP).

## Data Availability

The genome sequence data of *Andreaea rupestris* that support the findings of this study are openly available in GenBank of NCBI at (https://www.ncbi.nlm.nih.gov/) under the Accession no. MW561627. The associated BioProject, Sequence Read Archive (SRA), and Biosample numbers are PRJNA699373, SRR13626418, and SAMN17774838, respectively.
